# Multiple periodicity in a nanoparticle-based single-electron transistor

**DOI:** 10.1038/s41467-017-00442-6

**Published:** 2017-09-01

**Authors:** O. Bitton, D. B. Gutman, R. Berkovits, A. Frydman

**Affiliations:** 1 Chemical Research Support department, Weizmann Institute of Science, Rehovot, 76100 Israel; 2 The Institute of Nanotechnology and Advanced Materials, The Department of Physics, Bar Ilan University, Ramat Gan, 52900 Israel

## Abstract

A single-electron transistor is a nano-device with large potential for low-power applications that can be used as logic elements in integrated circuits. In this device, the conductance oscillates with a well-defined period due to the Coulomb blockade effect. By using a unique technique, we explore single-electron transistors based on a single metallic nanoparticle with tunable coupling to electric leads. We demonstrate a unique regime in which the transistor is characterized by multi-periodic oscillations of the conductance with gate voltage where the additional periods are harmonics of the basic periodicity of the Coulomb blockade and their relative strength can be controllably tuned. These harmonics correspond to a charge change on the dot by a fraction of the electron charge. The presence of multiple harmonics makes these transistors potential elements in future miniaturization of nano-sized circuit elements.

## Abstract (web summary)

Single-electron transistors are elements for nanoscale electronics. Employing single-electron transistors based on gold nanoparticles, Bitton et al., report a fabrication technique that allows precise control over the coupling between a nanodot and leads, resulting in new transport characteristics.

## Introduction

Single-electron transistors (SETs) are based on a nanostructure such as a nanoparticle, molecule, or a quantum dot, which is resistively coupled to the source and drain leads and capacitively coupled to a gate electrode. Electrons are confined to a small volume and their number in the nanostructure is quantized. The current through the nanostructure can be tuned via the gate voltage which controls the number of electrons in the SET. Each time a single electron is added, the current is blocked due to Coulomb blockade (CB) effect^1–5^. Hence the device exhibits conductance oscillations as a function of gate voltage, *V*_g_, with a well-defined CB periodicity (*P*_CB_ = *e*/*C*_G_) equal to the ratio of an electron charge *e* to a gate capacitance *C*_G_. The periodic conductance oscillations make the SET a promising ground for applications such as an electronic switch^6^, memory device^7^, extremely sensitive charge and displacement sensor^8, 9^, logic gates^10, 11^, voltage amplifier^12^, and so on.

Applications of multi-input gate SETs for multiple-valued logic circuits have also been proposed^13^. This requires the fabrication of multi-SETs for each logic circuit. For instance, two multi-gate SETs can produce three current levels. A significant increase in SET functionality may be achieved if a single SET would be able to produce multiple well-distinguished current values between the high state (CB peak) and low state (CB valley). This would enable the use of a single SET for switching between more than two states and therefore would facilitate going beyond binary logic. In this case, a set of multiple inputs, all connected to the gate electrode of a single SET, would generate an output which can have a few different values. Such a device will not only allow easier access to a multiple-valued logic function but would also be a large step toward further miniaturization of integrated circuit elements.

In this work, we show that under certain conditions an SET can manifest multiple-periodicity where the relative intensity of each period can be well-controlled. A superposition of multiple periods having different intensities naturally yields different output values. The ability to manipulate the relative intensity of each period enables one to fine-control the output current values of the SET device.

Multiple-periodicity is not a typical characteristic of conventional SET devices in which the coupling between the nanostructure and any lead is weak and the conductance through each barrier is much smaller than the quantum conductance *e* ^2^/*h*. In this weak coupling regime, the charge confined in such a closed dot is quantized and one observes a well pronounced set of conductance peaks. Each peak is associated with the change of total charge on the dot by one electron. The other limit is the strong coupling regime for which at least one of the leads is open, with the coupling greater than *e* ^2^/*h*. In this open dot the charge is not quantized, CB effects are suppressed, and one observes only a weak modulation of the conductance through the dot, with the same period^5, 14^. The crossover between closed and open dots is seldom investigated. In recent theoretical works^15, 16^ the crossover regime was approached from an open dot limit (see Supplementary Discussion). It was shown that for chaotic dots (for which the electron explores the entire space of the nanoparticle)^17^, with a large number of weakly open channels, additional conductance vs. gate voltage oscillations emerge with periods that are equal to the base *P*_CB_, divided by an integer number *n*. These are clearly detected using Fourier transform analysis as multiple harmonics *f*_*n*_ = *n*/*P*_CB_. Such oscillations correspond to a change of the charge on the dot by *e*/*n*. Of course, this does not violate charge quantization, but rather means that in the strong coupling regime the electronic wave function is split between inside and outside the dot. Since the CB effect is sensitive only to the charge confined inside the dot the conductance oscillates with a fractional charge periodicity. Remarkably, chaotic dynamics gives rise only to integer fractions of *P*_CB_, as indeed clearly seen in our experiment.

Over the past few decades several experimental methods have been used to fabricate SETs, most of which cannot fulfill the predicted conditions for additional periodicities in the conductance. Conventional SETs are based on low-carrier-density two-dimensional electron gases in which opening the dot is achieved by applying back gate voltages^2–5, 18^, hence making it more likely to yield very few channels which are well connected to the leads rather than many weakly connected channels. Other fabrication methods include electromigration^19, 20^, mechanically controlled break junction^21^, angle evaporation^22, 23^, chemically assembled SETs^11, 24^, and synthesis of a dimer structure^25^. Within the framework of these works many attempts have been taken to reduce the tunneling barriers in SETs devices in order to improve its signal-to-noise ratio and functionality. In some of these works the strong coupling regime has been reached and studied^22^. Nevertheless the ability to systematically control the coupling within the regime where the quantum conductance is *e* ^2^/*h* or larger, remains challenging.

Here, we present results on a unique set-up of an SET based on a metallic nanoparticle with controllable coupling to a set of leads. We show that for a relatively open dot the conductance vs. gate voltage features multi-periodic structure which can be controlled either by coupling strength or bias voltage.

## Results

### Experimental set-up of the SET system

**Fig. 1:**
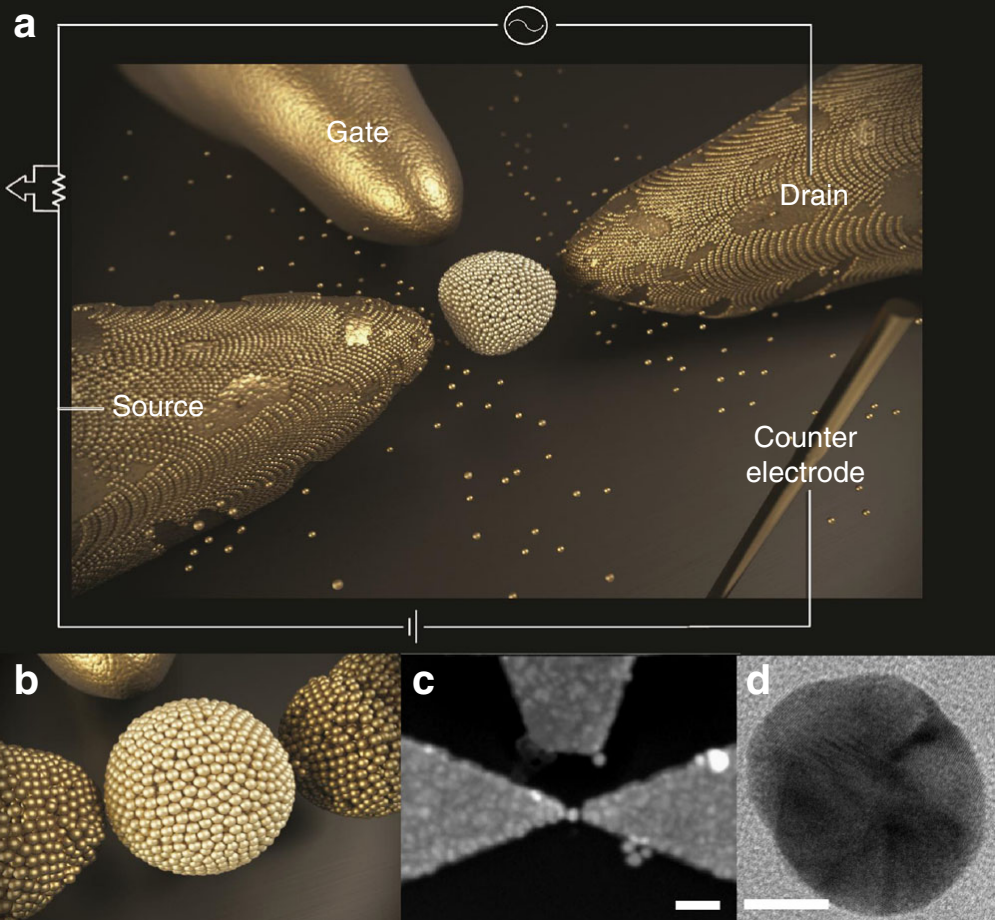
The fabrication system. **a** An illustration of the nanoparticle and the arrangement of source, drain and gate electrodes. The device is immersed in the electrodeposition solution and gold atoms are grown on the source and drain electrodes thus closing the gap between them and the nanoparticle. **b** A closeup of the single-electron transistor demonstrating the expected configuration after the electrodeposition process in which the particle is coupled to the leads via a number of close atoms. **c** Scanning electron microscope image of a dot-leads system. The *scale bar* represents 100 nm. **d** Transmission electron microscope image of a gold colloid. The *scale bar* represents 10 nm

The SETs are prepared utilizing a unique technique that combines nanolithography, atomic force microscope (AFM) nanomanipulation and electrodeposition as illustrated in Fig. 1 and described in the Methods section. Electrodeposition techniques have been used in the past to control the gap between two electrodes^26–28^. We take this method one step further and construct controllable SETs that are based on chaotic metallic nanoparticles^29, 30^. These SETs fulfill the theoretical conditions required for observing multi-periodicity. First, the nanoparticle contains a set of crystallographic facets as is clear from the transmission electron microscope (TEM) image of Fig. 1d, thus rendering the dot chaotic. More importantly, these devices are naturally characterized by multi-channel coupling since coupling is achieved via a set of atoms which couple in parallel to the Au nanoparticle (see Methods section and Fig. 1b). Finally, the technique enables one to fine-tune the system to the right dot-lead coupling strength which is relevant for CB harmonics to be measurable.

### Appearance of higher harmonics

**Fig. 2:**
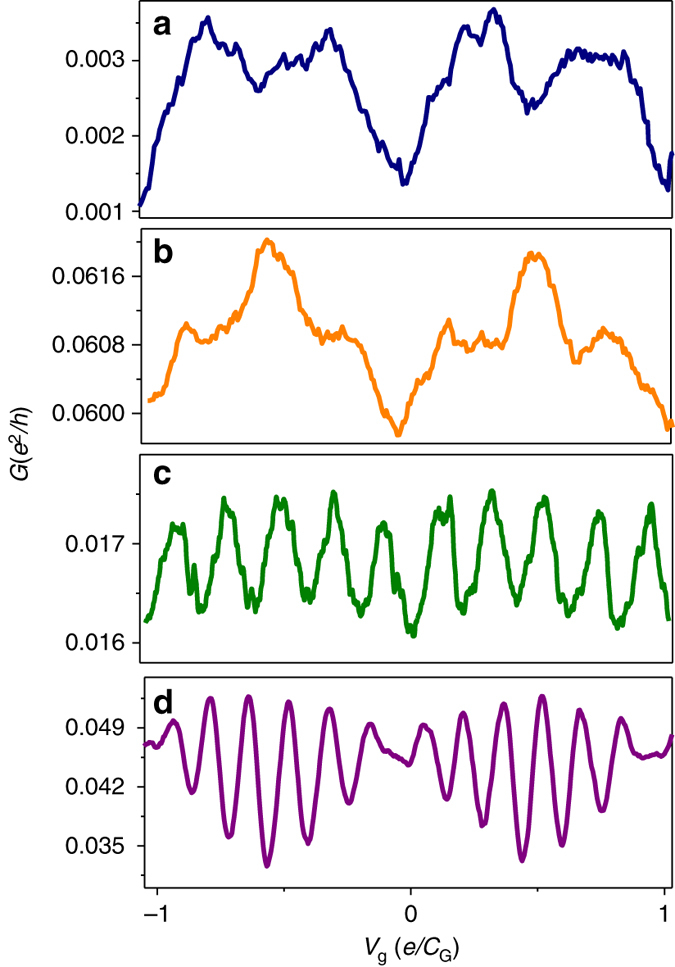
Multiple harmonics. *G*(*V*_g_) for four different single-electron transistors. All *curves* show conductance oscillations with a periodicity *P*_CB_, and additional oscillations with frequencies that are the second **a**, third **b**, fifth **c**, and sixth **d** harmonics of *f*_CB_ = 1/*P*_CB_. All curves were taken for *V*_SD_ = 1 mV

A typical conductance vs. gate voltage curve *G*(*V*_g_) of such an SET system taken at *T* = 4.2 K is shown in Fig. 2a. The curve exhibits two clear periods, one is the base *P*_CB_ and the other is half this period. We measured CB effects on 21 similar samples. Multiple-periodicity was observed in nine such SET devices. In most cases the SETs exhibit conductance oscillation with the base period *P*_CB_ and additional oscillation with a period *P*_CB_/2. However, some SETs exhibit oscillations that give rise to the third (Fig. 2b), fifth (Fig. 2c), and sixth (Fig. 2d) harmonics of the CB frequency *f*_CB_ = 1/*P*_CB_.

**Fig. 3:**
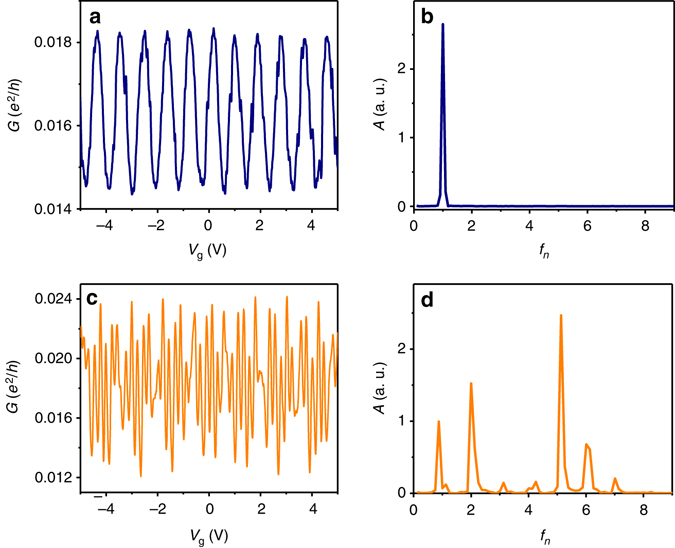
Effect of coupling on the multiple-periodicity. **a** *G*(*V*_g_) for a strongly coupled dot (*η* = 7.7 ± 0.1) and its Fourier transform **b**. **c** *G*(*V*_g_) for a weaker coupled dot (*η* = 3.3 ± 0.1). **d** The Fourier transform of **c** showing seven different harmonics (the *x*-axis is in units of the base Coulomb blockade frequency *f*_CB_). Both measurements were taken for *V*_SD_ = 1 mV. We note that the reason that the stronger coupled dot has a smaller conductance is the asymmetrical coupling to the drain and source (see main text)

It turns out that the intensity of the faster periods is very sensitive to the dot-lead coupling strength *η* = (1/*R*_S_ + 1/*R*_D_)*h*/*e* ^2^, controlled by the resistances between the dot and both source (*R*_S_) and drain (*R*_D_) electrodes. We only observe multiple-periodicity for SETs which are tuned to the appropriate coupling regime. Unfortunately, it is not possible to measure the coupling strength directly from the conductance. Moreover, since our dot is asymmetrically coupled, these two quantities are quite different. While the measured conductance, *G*, is governed by the weakly connected lead, the coupling strength, *η* is determined by the well connected one. Nevertheless, it is possible to extract *η* by fitting *I*−*V* curves to the results of ref. ^31^ as discussed in the Supplementary Discussion (see also Supplementary Fig. 2 and ref. ^30^). Doing so we find that there is a small coupling window where multiple-periodicity can be observed, that lies in the interval 2 < *η* < 5. This is demonstrated in Fig. 3 which depicts conductance vs. gate voltage curves *G*(*V*_g_) for two SETs with two different coupling strengths. In Fig. 3a the dot is strongly coupled (*η* = 7.7 ± 0.1) and the conductance oscillates with a base *P*_CB_ only, as demonstrated in the Fourier transform (Fig. 3b). In Fig. 3c, on the other hand, the dot is weaker coupled (the coupling strength is smaller by about a factor of two, *η* = 3.3 ± 0.1) and the conductance exhibits additional periods. In this case, the structure is much richer than those of Fig. 2. The Fourier transform depicted in Fig. 3d reveals that the conductance curve is composed of seven well-defined periodicities which are identified as harmonics of the basic *f*_CB_.

**Fig. 4:**
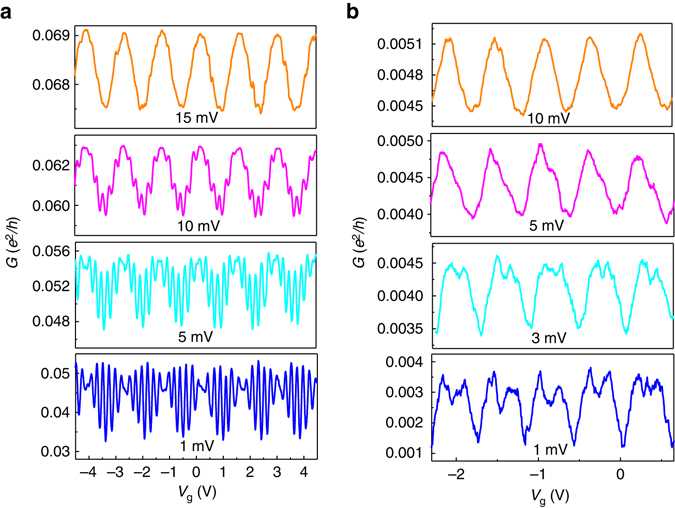
Effect of bias voltage on the multiple-periodicity. *G*(*V*_g_) for two different dots at different values of bias voltage. For both dots, at *V*_SD_ = 1 mV two dominant periods are clearly observed. For higher values of bias voltage (*V*_SD_ > 10 mV) a period corresponding to the base *P*_CB_ is dominant. **a** The dot is characterized by *η* = 4.7 ± 0.1. The ratio between the two dominant periods is 6. **b** The dot is characterized by *η* = 3.4 ± 0.1. The ratio between the two periods is 2

Interestingly, it is seen that the relative amplitude of the different harmonics is not monotonic with the harmonic order. In this case the second harmonic has a larger amplitude than the first harmonic and the fifth harmonic is the most prominent. Similar Fourier analysis on Figs. 2c and 4a reveal that the intensity of the high harmonic is larger than that of *f*_CB_. Though the theory predicts (Supplementary Discussion) that the occurrence of additional harmonics in the conductance is a generic property of open dots, and accounts for the *I*−*V* characteristics and the magnetic field dependence (Supplementary Fig. 3), it does not explain enhanced amplitudes of high harmonics observed in some of our samples. Because this feature is sample dependent it is reasonable to attribute it to the stochastic nature of our dots. It is quite plausible that some of the dots are not fully ergodic, and cannot be accounted for by random matrix theory. In this case one expects large sample to sample fluctuations of the harmonic strength that are sensitive to the details of dot-lead coupling and cannot be computed analytically.

### Bias dependence

A major advantage of our devices is that the strength of the different periods can be tuned not only by coupling strength but also by bias voltage. It turns out that, for any coupling strength, as the bias voltage is increased, the higher harmonics are suppressed. The mechanism behind this suppression are non-equilibrium current fluctuations that destroy quantum coherence. This suppresses higher harmonics stronger than the lower ones, as demonstrated in Fig. 4 for two different dots measured at different values of bias voltage. While for small bias voltage an extra harmonic is clearly observed in the conductance curves, at higher *V*_SD_ this harmonic is unmeasurable and only the base *f*_CB_ is observed. Therefore by scanning the bias voltage, one can tune the relative strength of the periodicities and control the values of output current of an SET. The fact that the additional harmonics can be switched on and off by bias voltage makes these SETs very appealing for device applications.

In summary, we have demonstrated an SET device, which incorporates multi-periodic conductance oscillations. The relative strength of the periods can be tuned by either the dot-lead coupling or by the bias voltage making this type of device very favorable for integration in electronic circuits. The conditions for such multiple-periodicity are clearly listed and fulfilled by our unique fabrication process which enables one to tune the coupling strength to a regime in which the charging of the dot corresponds to a fraction of an electron charge.

## Methods

### Fabrication of Au electrodes

The first step in device fabrication is the formation of source, drain, and gate Au electrodes on a Si–SiO substrate. This is achieved by a combination of photo-lithography and e-beam lithography (RAITH, Elphy Quantum) for fabrication of source and drain electrodes separated by a small gap of 10–30 nm and a side gate electrode at a distance of 200 nm.

### Nanomanipulation of Au nanoparticle

The nanoparticles we use are gold colloids with diameter of 30 nm. We use colloidal particles in solution which are stabilized by negatively charged ions that prevent agglomeration of the particles in solution. By using an organic layer that is terminated with amino groups, it is possible to adsorb the negative shell of the particles to the substrate by electrostatic interaction. We chose Poly-L-Lysine (P.L.L) as an adhesive layer. After the deposition of the adhesive layer the sample is ready for the deposition of the gold colloids. The Au colloid deposition takes place by adding a 10–20 μl drop of the solution on the modified substrate for an hour. This results in 20–30 gold colloids within a 1 μm square area.

For placing the nanoparticle in a desired location we utilize AFM nanomanipulation (Nanoman system in DI Veeco 3100 Scanning Probe Microscope). The nanoparticle is moved between the electrodes using the AFM tip, by pushing the particle to the right position.

### Electrodeposition

After positioning the colloid in the gap, the distance to each lead is usually smaller than 10 nm; however, the dot is not yet electrically connected to the leads. At this stage, we are not able to monitor current through the device. For minimizing the gap between the nanoparticle and the leads, we use an electrodeposition process by which we grow atoms on the leads. During the deposition process, we measure the conductance between the source and drain and stop the process when current can be measured. For the electrodeposition process, we place the sample on a holder which is made of inert materials (glass and Teflon). This is very important for preventing any metal dissolution that may result from reaction with the solution. The electrodes are connected to the circuit through Cu wires pressed by Indium which are placed out of the solution.

Our electrodeposition set-up consists of solution, counter electrode, reference electrode, and working electrodes (Fig. 1a). The electrolyte is an aqueous solution consisting of 0.01 M of potassium cyanurate (KAu(CN)_2_) and a buffer (PH 10) composed of 1 M potassium bicarbonate (KHCO_3_) and 0.2 M potassium hydroxide (KOH). A 25 mm diameter Au wire (99.9985% purity) is used as the counter electrode and the reference electrode. The two separated evaporated gold electrodes are the working electrodes. When applying electrochemical DC voltage between the working electrodes and the counter electrode, the cyanurate ion accepts an electron from the working electrodes and liberates the cyanide ligands. Hence, neutral gold atoms collect on the surface of the two gold electrodes and close the gap between the leads and the dot.

The deposition occurs only on the electrodes and not on the colloid as it is not connected to the circuit. Deposition occurs on the colloid only after it becomes electrically connected to one of the electrodes and becomes part of it.

The value of the DC voltage between the counter electrode and the source and drain electrodes determines the deposition rate. The slower the deposition, the higher is the quality of the film. We found that a value of 1 V yields a slow enough deposition rate of 0.3 nm min^−1^. Under these conditions the deposition is very uniform and smooth, thus the possibility of large gold clusters forming during the electrodeposition process becomes improbable. In addition, the slow rate enables to stop the process at different degrees of couplings.

The deposition is isotropic, hence the gold is deposited uniformly on the surface of the electrodes with no preference to a certain direction. Therefore, bridging the dot-leads distance causing the widening of the electrodes tips. It is for this reason that the coupling between the dot to the leads and hence the dot-lead conductance is believed to be achieved by a large number of channels.

**Fig. 5:**
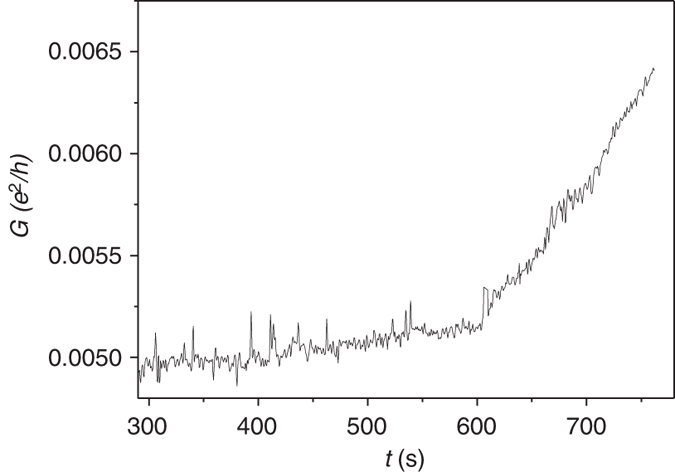
Conductance measurement during electrodeposition. Dimensionless conductance as a function of time of a dot-leads system measured during the electrodeposition process

For measuring the conductance between the working electrodes during the electrodeposition process, we use AC conductivity measurement which ensures equal deposition on both the electrodes. An AC voltage of 2 mV is applied between the two working gold electrodes. We are able to monitor the separation between the electrodes once the distance becomes very small. The oscillation frequency should be very low in order to reduce the ion conductivity of the solution which appears as a background measurement. Applying frequency of 2 Hz reduces the measured conductivity of the solution to 5 MΩ. This value is always measured at the background. Therefore it is extremely difficult to stop the process at the stage of a very weakly connected barrier. Figure 5 shows a conductance as a function of time of a dot-lead system during the deposition process. It is seen that after 10 min of deposition the conductance starts to increase indicating that the dot-lead barriers have conductances comparable to the solution background conductance.

**Fig. 6:**
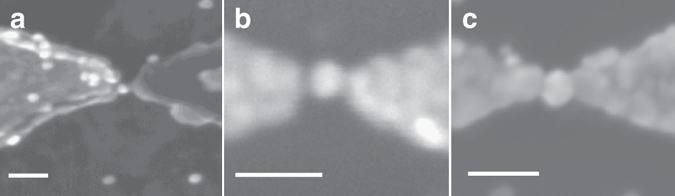
Scanning electron microscope (SEM) images of different coupled systems. SEM images of samples after: **a** Trapping a gold nanoparticle into the gap. **b** Applying the electrodeposition process but before any current could be measured through the dot. **c** After the sample was fully connected by electrodeposition and the dot becomes part of the electrodes. All *white scale bars* represent 100 nm

After electric contact is achieved the sample is taken out of the aqueous solution and transferred to a measurement probe where it is cooled down to 4.2 K for electrical measurements. High resolution scanning electron microscope (SEM) images of different samples taken after different fabrication stages are shown in Fig. 6.

The success of the electrodeposition process crucially depends on the symmetry of the distance between the dot and the leads. If the metallic colloid is not positioned at the center of the gap, there is a very small chance to stop the electrodeposition process at measured conductance of *G* ≤ *e* ^2^/*h* while still being able to observe CB effects. Twenty-five percent of our electrodeposited samples showed CB effects while the rest showed ohmic conductance curves, apparently due to strong antisymmetric geometry. An example of such asymmetric device is shown in Fig. 6c. In this case, when measurable current appeared through the device, the dot was already fully connected to the right electrode, and therefore no CB effects were resolved.

Finally, it is important to note that the same fabrication technique, including the adsorption of the adhesive layer, P.L.L, was applied to several devices without placing a nanoparticle in the gap. All these devices did not show any signature of CB effects in the conductance curves (Supplementary Discussion and Supplementary Fig. 1). This assures that parasitic junctions or dots are not formed during the fabrication process.

### Data availability

The data that support the findings of this study are available from the corresponding author on reasonable request.

## Electronic supplementary material

Supplementary Information

Peer Review File
