# Supplementary material for: Multiple periodicity in a nanoparticle-based single-electron transistor
Source: Nat Commun. 2017 Sep 1;8:402. doi: 10.1038/s41467-017-00442-6 (PMC5581334; doi:10.1038/s41467-017-00442-6)
Supplement: Supplementary file 1 — Supplementary Information [file 41467_2017_442_MOESM1_ESM.pdf]

Description of Supplementary Files

File Name: Supplementary Information
Description: Supplementary Figures, Supplementary Discussion and Supplementary References

File Name: Peer Review File

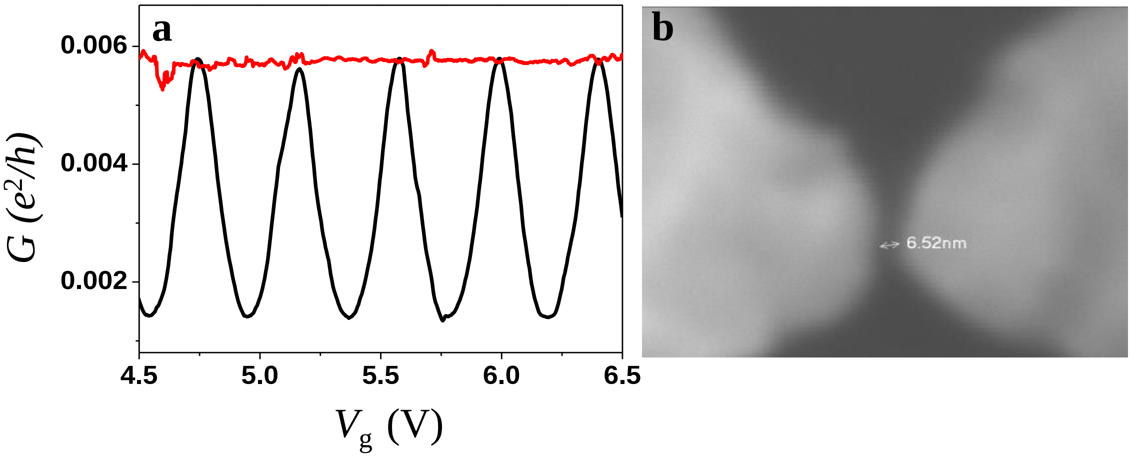

Supplementary Figure 1: Conductance curves for samples with and without a colloid. **a.** Two conductance curves as a function of gate voltage taken at V_{SD}=1 mV for two samples. One with a trapped colloid (black curve) and the other without a colloid (red curve). **b.** Scanning electron microscope image of the system without the dot for which the red curve measurement (in **(a)**) was taken after the electrodeposition process. White scale bar represents 10 nm.

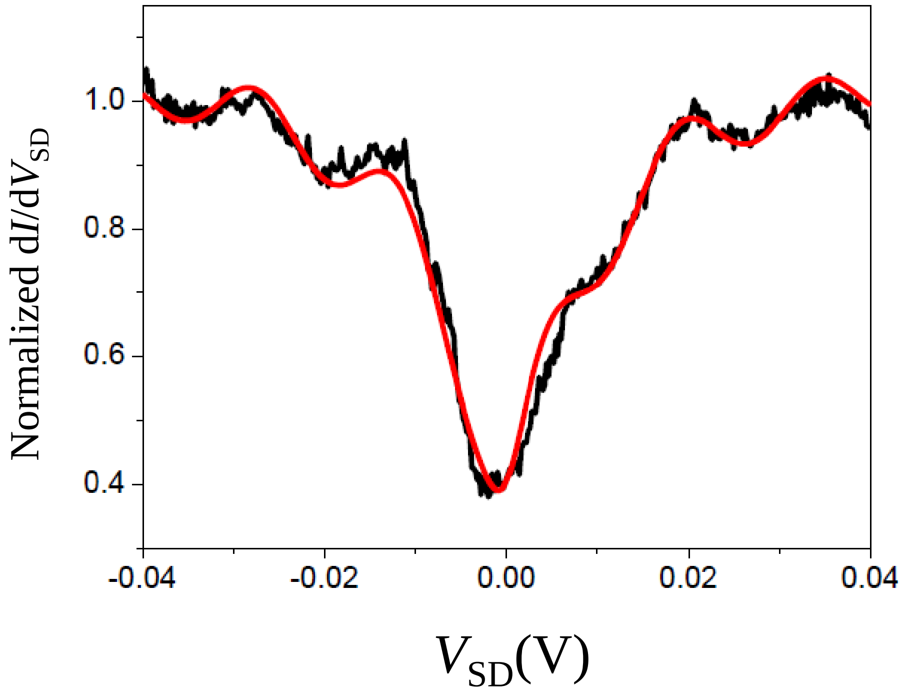

Supplementary Figure 2: Conductance as a function of bias voltage. Differential conductance as a function of bias voltage for a gate voltage of 0.3 V. The red curve is a fit to Eq. (4) of Supplementary Discussion.

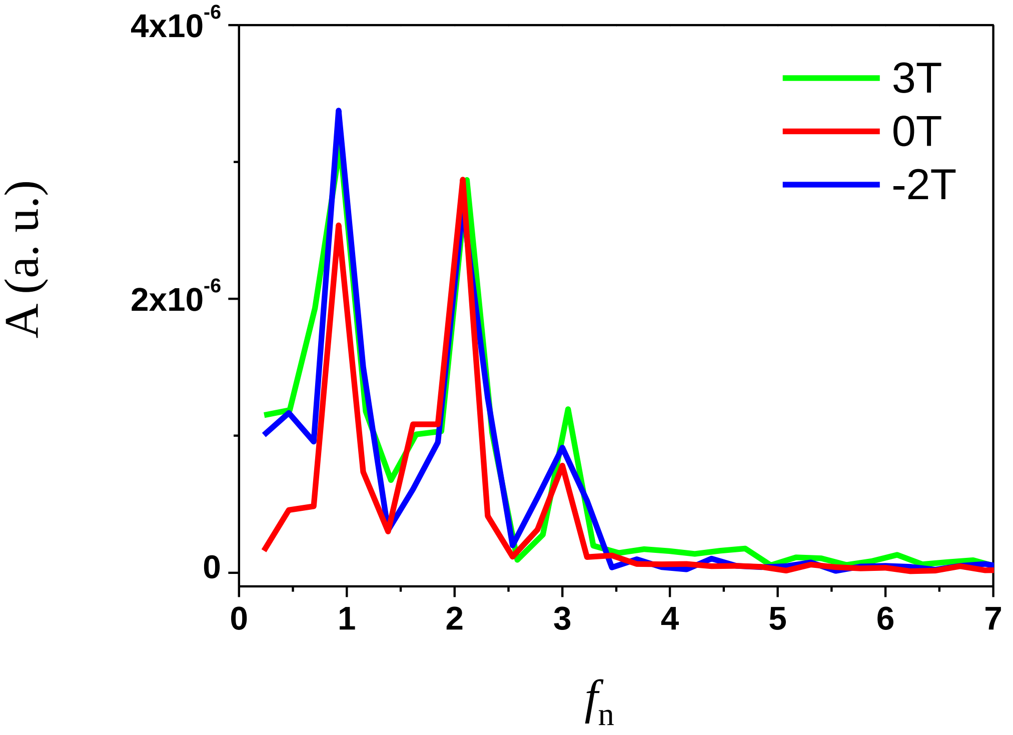

Supplementary Figure 3: Magnetic field dependence. Fourier transform of $G(V_{\mathrm{g}})$ for an SET system at different values of magnetic field with a range corresponding to one flux quantum penetrating the dot (5 T). The x-axis is in units of f_{CB}.

Supplementary Discussion

Multiple periods may also appear in setups with several capacitively coupled dots. Such a scenario is not consistent with our results. First, the CB staircases of our samples show only a single period. This is inconsistent with a multiple dot configuration. Secondly, all the additional harmonics observed in the experiment are found to be multiples of f_{CB}. It is highly unlikely that such a correlation is accidental. Moreover, we performed scanning electron microscope (SEM) and atomic force microscope (AFM) imaging for various stages of the fabrication process. All images clearly show a *single* nanoparticle controllably placed in the gap between the leads. Examples are shown in Figs. 1C and 6 in the manuscript.

To rule out the possibility that parasitic Au clusters form close to the prime dot during the deposition process, we applied a similar electrodeposition process on samples without a dot in the gap. Supplementary Fig. 1 compares the $G(V_{\mathrm{g}})$ curves for setups with and without the Au colloid. While for the former case CB oscillations are clearly observed (black line), the $G(V_{\mathrm{g}})$ is practically constant for the latter case (red curve). Note, that the two samples have a similar conductance value. This shows that no unintentional junctions form during the electrodeposition process. In addition, chemical analysis reveals that the gold atoms are evaporated only on the electrodes and not on the SiO substrate thus excluding the possibility on an additional Au particle growth. We, therefore, conclude that the cause for the multiple periods is an inherent property of a single nanoparticle.

As we show below such a behavior naturally follows from a standard model of a chaotic dot[1], connected to electrodes by a large number of low transparency channels. At high temperatures the source-drain resistance (R) is purely classical and given by the sum of source-dot (R_{S}) and drain-dot (R_{D}) resistances, $R = R_{\mathrm{S}} + R_{\mathrm{D}}$. We focus on the strongly coupled dot, where the dimensionless coupling strength $\eta = (1/R_{\mathrm{S}} + 1/R_{\mathrm{D}})h/e^2$ is large ($\eta \gg 1$). Note, that the coupling strength η differs from the dimensionless source-drain conductance $h/e^2 R^{-1}$. While the source-drain conductance vanishes for the dot coupled to one lead only, its coupling strength remains finite.

Since the charge inside the dot strongly fluctuates, it is convenient to use a canonically conjugated variable - phase ϕ [2]. The quantum dot is thus described by a "dissipative action"[2–6]. At equilibrium, at temperature T, it is governed by imaginary time action

$$S = \frac{\eta T^2}{4} \int_0^{1/T} d\tau_1 d\tau_2 \frac{e^{i\phi(\tau_1) - i\phi(\tau_2)}}{\sinh^2 \pi T(\tau_1 - \tau_2)} + \frac{1}{4E_\mathrm{c}} \int_0^{1/T} d\tau \dot{\phi}^2 - iq \int_0^{1/T} d\tau \dot{\phi} \,. \tag{1}$$

Here $E_\mathrm{c} = \frac{e^2}{2C}$ is the charging energy of the dot where C is the capacitance of the dot that is determined by capacitive coupling to the source, the drain, and the gate, $C = C_\mathrm{S} + C_\mathrm{D} + C_\mathrm{G}$. The average number of electrons on the dot is

$$q = \frac{C_\mathrm{S} R_\mathrm{S} - C_\mathrm{D} R_\mathrm{D}}{e(R_\mathrm{S} + R_\mathrm{D})} V_\mathrm{SD} + \frac{C_\mathrm{G}}{e} V_\mathrm{g} \,. \tag{2}$$

where V_SD is the bias voltage and V_g is the gate voltage. The action (1) has non-trivial minima

$$e^{i\phi(\tau)} = \prod_{n=1}^{|W|} \left(\frac{e^{2\pi i \tau} - z_n}{1 - \bar{z}_n e^{2\pi i \tau}} \right)^{\mathrm{sgn} W} , \tag{3}$$

known as Korshunov instantons [7]; z_n and $\bar{z}_n$ are global variables that determine the position and the size of the instanton; W is a winding number that counts the number of times the phase ϕ circles around the origin. Above a certain temperature (of the order $T_* \simeq E_\mathrm{c} \exp[-\eta]$) the instantons are rare, and they independently contribute to the source-drain conductance [6]

$$G(T) \simeq \frac{e^2}{h} \frac{R_\mathrm{D} R_\mathrm{S}}{(R_\mathrm{S} + R_\mathrm{D})^2} \left[\tilde{\eta} + \sum_{W=1}^{\infty} a_W e^{-F_W(T)} \cos(2\pi q W) \right] \tag{4}$$

where we define $a_W \simeq \eta^{W+1} \psi^{(m)}(1)/2\pi$, and ψ^m is the mth order derivative of the digamma function. For the open dot limit, the amplitude of harmonic number W is suppressed in accordance with Eq.(4), where the function

$$F_W(T) \simeq \frac{\tilde{\eta} W}{2} + \frac{\pi^2 T}{E_\mathrm{c}} W^2 \tag{5}$$

The first term in Eq.(4) corresponds to the topologically trivial part with the winding number $W = 0$. It accounts for zero bias anomaly (ZBA) due to renormalization of the coupling strength $\eta \to \tilde{\eta} = \eta + \ln(1 + \omega^2 t_\mathrm{c}^2)$ by quantum fluctuations around a trivial minima, in agreement with quantum Langevin approach [3]. As for the higher dimensional cases discussed [8–13], the dip magnitude is inversely proportional to η but always pinned

to the Fermi energy, i.e., at $V_{\rm SD} = 0$. Here the infrared energy scale $\omega = \max(T, eV)$ and RC time $t_{\rm c} = \frac{R_{\rm S}R_{\rm D}}{R_{\rm S}+R_{\rm D}}C$.

By fitting the differential conductance curves as a function of bias voltage to Eq. (4) we can extract the values of the system's parameters [14]. In this procedure $R_{\rm S}$, $R_{\rm D}$ and $E_{\rm c}$ are used as fitting parameters, that determine η and $t_{\rm c}$. Supplementary Fig. 2 shows an example of a fit to a differential conductance curve of one of our samples, taken at a gate voltage of 0.3V and for $E_{\rm c}$ =16 mV. This fit yields $\eta = 3.5 \pm 0.1$. In this case, the measured conductance was $G = 0.005\frac{e^2}{h}$ and this confirms that the sample is strongly asymmetrically coupled where the resistance of the well connected barrier is $R_{\rm D} = 7.4k\Omega$. We note that we use the same η for all the terms of Eq. (4) thus increasing our confidence in the procedure.

The topologically non-trivial solutions give rise to higher harmonics in oscillations of conductance with the gate voltage. The instantons with the winding number W correspond to a harmonic W in Fourier analysis of conductance oscillations. In the open dot limit, only the first harmonic survives, leading to weak single period oscillation [15]. As the coupling of the dot decreases, a finite number of harmonics is observed. For η of the order unity an infinite number of harmonics appear with a parametrically equal magnitude, and the instanton expansion breaks down. This result merges with the one known for the strong CB regime [16], i.e. for $\eta \ll 1$. In this case, the conductance is

$$G \simeq (R_{\rm S} + R_{\rm D})^{-1} \frac{\Delta E/2T}{\sinh \Delta E/2T}\,, \tag{6}$$

where $\Delta E = 2E_{\rm c}(q - [q])$ is the deviation from the degeneracy point. At $T \ll E_{\rm c}$ it corresponds to the sequence of well resolved CB peaks, that in a Fourier space gives rise to a large number of harmonics with approximately same magnitude.

The calculations performed above are valid at equilibrium, which is not the case for our experiment. As it was shown recently [17], Korshunov instantons can be generalized to a non-equilibrium situation, provided the value of a source-drain voltage is smaller than the charging energy. In this case, the instanton solution was explicitly constructed in terms of hypergeometric functions. Importantly, the values of dissipative action on the instanton solution are determined solely by analytic property of the solution on the time plane, treated as a complex variable. Consequently, the value of the action on instanton trajectory out of the equilibrium is equal to the one at the thermal equilibrium. Therefore, with exponential accuracy, these results are robust to external bias. To fit the theory to mea-

surements, one needs to know not only the value of the exponent but also the prefactors. The latter account for fluctuations of the bosonic field around non-perturbative solution, and are influenced by non-equilibrium dephasing [18, 19]. To calculate them analytically, one needs to compute the fluctuation determinants in the vicinity of a non-perturbative solution. This is an extremely challenging task, and full mathematical analysis is yet to be done. To progress, we employ a phenomenological approach, proposed in Ref.[3], where the fluctuations around topologically non-trivial solution were approximated by ones near trivial vacuum. In this case the thermal smearing out of equilibrium is accounted for by $F_W(T) \to F_W(T) + (2\pi/e^2)W^2 \sum_{r=\mathrm{S,D}} y_r(x)/R_r$, where $x_r = R_\mathrm{S} R_\mathrm{D} R_r eVC/(R_\mathrm{S}+R_\mathrm{D})^2$, and $y(x) = x\arctan(x) - 1/2\ln(1+x^2)$.

Considered as a function of q, the contribution of the instanton with winding number $W = 1$ is periodic with the period unity, that corresponds to a unit charge. Indeed, such instanton accounts for the phase lapse by 2π that amounts to a pumping of one electron through the dot and gives rise to a basic periodicity P_CB in the thermodynamic potentials and transport coefficients. Instantons with a winding number W give rise to fractional periodicity P_CB/W that corresponds to a transfer of $1/W$ of the electron charge. This does not violate a charge quantization, since in an open dot any (not necessarily integer) charge can be redistributed between interior and exterior of the dot. Such processes lead to periodic dependences with respect to a fraction of electron charge. We believe this picture, i.e. the occurrence of additional harmonics in the conductance is a universal property of open dots, however, the weights of the harmonics are model specific. For a not-fully-chaotic dot the statistics of wave function is not universal, and there is a finite probability to find a strongly coupled state with an average charge corresponding to a fraction of an electron charge inside the dot. Since such states give rise to the pronounced oscillation with a corresponding harmonic, one expects large sample to sample fluctuations of the harmonic strength. This may be the reason why for part of our samples the strengths of certain high harmonics is higher than of the lower ones (see Figs. 2d, 3c,d and 4a in the manuscript). For the chaotic dot, on the other hand, the wave functions obey random matrix theory. In this case there is a good agreement between the theory and the experiment. This view is consistent with the observed magnetic field dependence.

Application of a magnetic field of the order of a flux quantum through the dot changes the single particle wave functions, thus affecting the relative magnitudes of different harmonics.

Indeed, the Fourier transform shown in Supplementary Fig. 3 for one of our dots reveals that the relative strengths of various harmonics oscillate with the magnetic field. One notes that the dominant harmonic changes from the first (for $B = -2$ T) to the second (at $B = 0$ T) and back to the first (at $B = 3$ T). The periodicity range of 5 T corresponds to a single flux quantum penetrating the dot. Within the dissipative action theory, the temperature/bias suppresses the higher harmonics stronger than the lower ones. It results from the two effects acting together: (a) the thermal broadening of the instanton contribution, Eq.(5), is multiplied by the winding number square (W^2T/E_c); (b) the zero bias anomaly leading to the logarithmic renormalization of $\tilde{\eta}$ is weakened at high temperatures [15, 20] (compared with the scale $t_{\rm c}$), and the terms $\tilde{\eta}W$ increases with temperature. Therefore increasing temperature (or voltage) suppresses the harmonics with higher winding numbers stronger than those with the lower ones. This behavior agrees with our measurements.

Supplementary References

[1] Alhassid, Y. The statistical theory of quantum dots. Rev. Mod. Phys. **72,** 895 (2000).

[2] Schön, G. & Zaikin, A. D. Quantum coherent effects, phase transitions, and the dissipative dynamics of ultra small tunnel junctions. Phys. Rep. **198,** 237 (1990).

[3] Golubev, D. S., König, J., Schoeller, H., Schön, G. & Zaikin, A. D. Strong Electron Tunneling through Mesoscopic Metallic Grains. Phys. Rev. B **56,** 15782 (1997).

[4] Burmistrov, I. S. & Pruisken, A. M. M. The problem of "macroscopic charge quantization" in single-electron devices. Phys. Rev. B **81,** 085428 (2010).

[5] Nazarov, Y. V. Coulomb Blockade without Tunnel Junctions. Phys. Rev. Lett. **82,** 1245 (1999).

[6] Altland, A., Glazman, L. I, Kamenev, A. & Meyer, J. S. Inelastic electron transport in granular arrays. Ann. Phys. (N.Y.) **321,** 2566 (2006) and references therein.

[7] Korshunov S. E. Coherent and incoherent tunneling in a Josephson junction with a "periodic" dissipation. JETP Lett. **45,** 434 (1987).

[8] Finkelstein, A. M. Influence of Coulomb interaction on the properties of disordered metals. Sov. Phys. JETP **57,** 97-108 (1983).

[9] Finkelstein, A. M. Metalinsulator transition in a disordered system. Sov. Phys. JETP **59,** 212-219 (1984).

[10] Finkelstein, A. M. Electron Liquid in Disordered Conductors. Sov. Sci. Rev. A **14 (2),** 1-101 (1990).

[11] Altshuler, B. L. & Aronov, A. G. Zero bias anomaly in tunnel resistance and electron-electron interaction. Solid State Commun. **30,** 115-117 (1979).

[12] Altshuler, B. L. & Aronov, A. G. Contribution to the theory of disordered metals in strongly doped semiconductors. Sov. Phys. JETP **50,** 968 (1979).

[13] Altshuler, B. L., Aronov, A. G. & Lee, P. A. Interaction Effects in Disordered Fermi Systems in Two Dimensions. Phys. Rev. Lett. **44,** 1288 (1980).

[14] Bitton, L., Gutman, D. B, Berkovits, R. & Frydman, A. Coexistence of Coulomb blockade and zero bias anomaly in a strongly coupled quantum dot. Phys. Rev. Lett. **106,** 016803 (2011).

[15] Goeppert, G. & Grabert, H. Single Electron Tunneling at Large Conductance: The Semiclassical Approach. Eur. Phys. J. B **16,** 687 (2000).

[16] Aleiner, I. L., Brouwer, P. W. & Glazman, L. I. Quantum Effects in Coulomb Blockade. Physics Reports **358,** 309 (2002).

[17] Titov, M. & Gutman, D. B. Korshunov instantons out of equilibrium. Phys. Rev. B. **93**, 155428 (2016).

[18] Altland, A. & Egger, R. Nonequilibrium dephasing in Coulomb blockade quantum dots. Phys. Rev. Lett. **102,** 026805 (2009).

[19] Gutman, D. B., Gefen, Y.& Mirlin, A. D. Zero bias anomaly out of equilibrium. Phys. Rev. Lett. **100,** 086801 (2008).

[20] Panyukov, S. V. & Zaikin, A. D. Charge Disorder in Granular Metallic Films. Phys. Rev. Lett. **67,** 3168 (1991).
