# Supplementary material for: Multiple periodicity in a nanoparticle-based single-electron transistor
Source: Nat Commun. 2017 Sep 1;8:402. doi: 10.1038/s41467-017-00442-6 (PMC5581334; doi:10.1038/s41467-017-00442-6)
Supplement: Supplementary file 2 — Peer Review File [file 41467_2017_442_MOESM2_ESM.pdf]

Editorial Note: this manuscript has been previously reviewed at another journal that is not operating a transparent peer review scheme. This document only contains reviewer comments and rebuttal letters for versions considered at Nature Communications.

Reviewers' comments:

Reviewer #4 (Remarks to the Author):

As mentioned in my previous report for Nature Nanotechnology, the authors present, probably for the first time, an experimental observation of higher harmonics in Coulomb-blockade like conductance oscillations as a function of gate voltage. Such a result should be of interest for the community of quantum transport and Nanoelectronics.

The authors have responded to my comments, and I find their responses satisfactory. Moreover, they have improved the paper, mainly by modifying Fig. 2, thereby avoiding a possible confusion of the reader.

Also, the research is clearly of fundamental nature and still far from a technological realization. It therefore seems to me that a publication in Nature Communications is more appropriate than in Nature Nanotechnology.

In my view the paper can now be published in Nature Communications.
